# Live cell imaging techniques to study T cell trafficking across the blood-brain barrier *in vitro* and *in vivo*

**DOI:** 10.1186/2045-8118-10-7

**Published:** 2013-01-21

**Authors:** Caroline Coisne, Ruth Lyck, Britta Engelhardt

**Affiliations:** 1 Theodor Kocher Institute, University of Bern, Bern, 3012, Switzerland

**Keywords:** BBB, Immune cell trafficking, Live cell imaging, CD4 T cells, CD8 T cells, α4-integrins

## Abstract

**Background:**

The central nervous system (CNS) is an immunologically privileged site to which access for circulating immune cells is tightly controlled by the endothelial blood–brain barrier (BBB) located in CNS microvessels. Under physiological conditions immune cell migration across the BBB is low. However, in neuroinflammatory diseases such as multiple sclerosis, many immune cells can cross the BBB and cause neurological symptoms. Extravasation of circulating immune cells is a multi-step process that is regulated by the sequential interaction of different adhesion and signaling molecules on the immune cells and on the endothelium. The specialized barrier characteristics of the BBB, therefore, imply the existence of unique mechanisms for immune cell migration across the BBB.

**Methods and design:**

An *in vitro* mouse BBB model maintaining physiological barrier characteristics in a flow chamber and combined with high magnification live cell imaging, has been established. This model enables the molecular mechanisms involved in the multi-step extravasation of T cells across the *in vitro* BBB, to be defined with high-throughput analyses. Subsequently these mechanisms have been verified *in vivo* using a limited number of experimental animals and a spinal cord window surgical technique. The window enables live observation of the dynamic interaction between T cells and spinal cord microvessels under physiological and pathological conditions using real time epifluorescence intravital imaging. These *in vitro* and *in vivo* live cell imaging methods have shown that the BBB endothelium possesses unique and specialized mechanisms involved in the multi-step T cell migration across this endothelial barrier under physiological flow. The initial T cell interaction with the endothelium is either mediated by T cell capture or by T cell rolling. Arrest follows, and then T cells polarize and especially CD4^+^ T cells crawl over long distances against the direction of flow to find the rare sites permissive for diapedesis through the endothelium.

**Discussion:**

The sequential use of *in vitro* and *in vivo* live cell imaging of T cells interacting with the BBB allows us to delineate the kinetics and molecular determinants involved in multistep extravasation of encephalitogenic T cells across the BBB.

## Background

The endothelial blood–brain barrier (BBB) protects the central nervous system (CNS) from the constantly changing milieu in the vascular compartment by strictly controlling the movement of molecules across its interface. Thus, the BBB also establishes the border between the immune system and the CNS. Immunosurveillance of the CNS is achieved by allowing defined immune cells that hold the specific molecular keys to breach the BBB and to enter the perivascular or leptomeningeal spaces [1]. Mechanisms operating at the BBB are, therefore, instrumental in controlling immune cell migration into the CNS. Whereas under physiological conditions the number of immune cells crossing the BBB is low, during CNS inflammation such as in multiple sclerosis (MS) or its animal model, experimental autoimmune encephalomyelitis (EAE), a high number of immune cells enter the CNS parenchyma causing inflammation, edema and demyelination [2]. Interestingly, even in the inflammatory state the BBB still controls immune cell migration into the CNS. This is exemplified by the fact that in MS and EAE, myeloid cells and activated memory/effector T cells preferentially cross the BBB. Thus, the kinetics and molecular interactions occurring between circulating immune cells with the BBB are crucial in the pathogenesis of EAE and MS.

In general, the multi-step recruitment of circulating immune cells across the BBB is regulated by the sequential interaction of various adhesion or signaling molecules on the leukocyte and on the endothelial cell surfaces [3,4]. First, the interaction of adhesion molecules from the selectin family with their cognate carbohydrate ligands induces the rolling of the immune cell along the endothelial cell surface at reduced velocity. Next, chemokines displayed on the endothelial surface bind to their respective G-protein coupled receptors (GPCRs) on the leukocyte. This triggers the activation of integrins on the immune cell surface via a conformational change. Activated integrins bind to their endothelial ligands of the immunoglobulin superfamily and mediate the firm arrest of the immune cell on the endothelial surface. The arrested immune cell polarizes and starts crawling on the endothelial surface in search of a site permissive for diapedesis. Successful migration of a circulating immune cell across the endothelial cell wall, therefore, requires the productive interaction of the immune cell with the endothelial cells at each step of the multi-step recruitment cascade [4]. Because the BBB endothelium is highly specialized, unique dynamics and molecular mechanisms are required for immune cell migration into the CNS.

Recently available sophisticated live cell imaging technologies combined with *in vivo* surgical window preparations that overcome anatomical barriers and with BBB models in flow chambers *in vitro*, have provided powerful tools to study the cellular and molecular mechanisms involved in immune cell migration under physiological and pathological conditions. Combining both techniques in the same laboratory ensures that the number of animals used is minimized.

Advantages for experiments with *in vitro* BBB models are high resolution imaging of the endothelium, easy molecular and biochemical manipulations, less variability and, last but not least, the possibility of a high throughput of experimental conditions. Using *in vitro* BBB models established from different genetically-modified mice, we defined the endothelial cell adhesion molecules mediating post-arrest T cell interactions and especially T cell crawling against flow on the BBB [5]. As these findings were confirmed by others *in vivo*[6], the *in vitro* flow chamber approach has proved meaningful. Nevertheless, the limitations of this experimental approach are the absence of blood viscosity and of pathophysiological flow conditions that occur *in vivo*. Thus verification of *in vitro* findings in experimental animals *in vivo* is advisable to overcome limitations of the *in vitro* system. Microscopic access to the CNS microcirculation for live cell imaging has been achieved by the development of sophisticated cranial and spinal cord window surgical preparations [7,8]. A cranial window allows direct visualization of the leptomeningeal and cortical grey matter microcirculation whereas a spinal cord window provides access to the leptomeningeal and spinal cord white matter microcirculation [9,10]. We have pioneered the use of epifluorescence intravital microscopy (IVM) of the spinal cord white matter microvasculature in the mouse to investigate in real time the molecular mechanisms involved in the multistep extravasation of CD4^+^ encephalitogenic T cells across the BBB *in vivo*[9,10]. These T cells induce experimental autoimmune encephalomyelitis (EAE), an animal model for multiple sclerosis (MS). Blocking T cell adhesion to the BBB by the functional blocking of α4-integrin, inhibits the development of EAE and is used as a therapeutic approach for the treatment of MS [10,11].

Our current insight into the molecular mechanisms involved in immune cell trafficking into the CNS relies on studies performed with CD4^+^ T cells in EAE. Accumulating evidence suggests, however, that CD8^+^ T cells are also critically involved in the pathogenesis of MS. Indeed, CD8^+^ T cells accumulate within active MS lesions, often outnumbering CD4^+^ T cells [12]. Therefore, in this study protocol we present our investigation on the multi-step recruitment of CD8^+^ T cells across inflamed spinal cord microvessels during EAE *in vivo*.

The aim here is to describe the *in vitro* and *in vivo* live cell imaging approaches we have used to study the dynamics and molecular mechanisms involved in the multi-step T cell migration across the inflamed BBB in the context of the animal model of MS. We will highlight the suitability of our *in vitro* imaging system of the BBB under flow for investigating the molecular mechanisms involved in mediating shear-resistant T cell arrest versus T cell crawling or T cell diapedesis across the BBB. In addition we will describe experimental procedures and results of studying the migration of CD8^+^ T cells across the inflamed BBB by means of intravital fluorescence videomicroscopy (IVM) of the spinal cord.

## Methods and design

### Live cell imaging of T cell recruitment across the BBB *in vitro*

#### CD4^+^ T cells

In this study we used the encephalitogenic proteolipid protein (PLP)_aa139-151_ specific CD4^+^ Th1 effector/memory T cell line, SJL.PLP7, that has been described in detail before [13]. T cells were used 3 days after the third or fourth re-stimulation with the PLP_aa139-151_ peptide at a concentration of 0.5 × 10^6^ cells per mL.

#### *In vitro* BBB models

The polyoma middle T oncogen immortalized mouse brain endothelioma cell line (bEnd5) was described in detail before [14,15]. The cells were used between passages 18 and 25 and cultured for at least 3 days on laminin-coated surfaces (Roche, Basel, Switzerland). The isolation and culture procedures of primary mouse brain microvascular endothelial cells (pMBMECs) have also been described in detail before [15-17]. These cells were cultured on Matrigel-coated surfaces (BD Biosciences, Allschwil, Switzerland) and used as primary cells (passage = 0) 5–7 days after plating. The yield of pMBMECs from one mouse brain suffices to seed 3 wells with a surface area of 0.3 cm^2^ each.

#### *In vitro* live cell imaging

**Figure 1:**
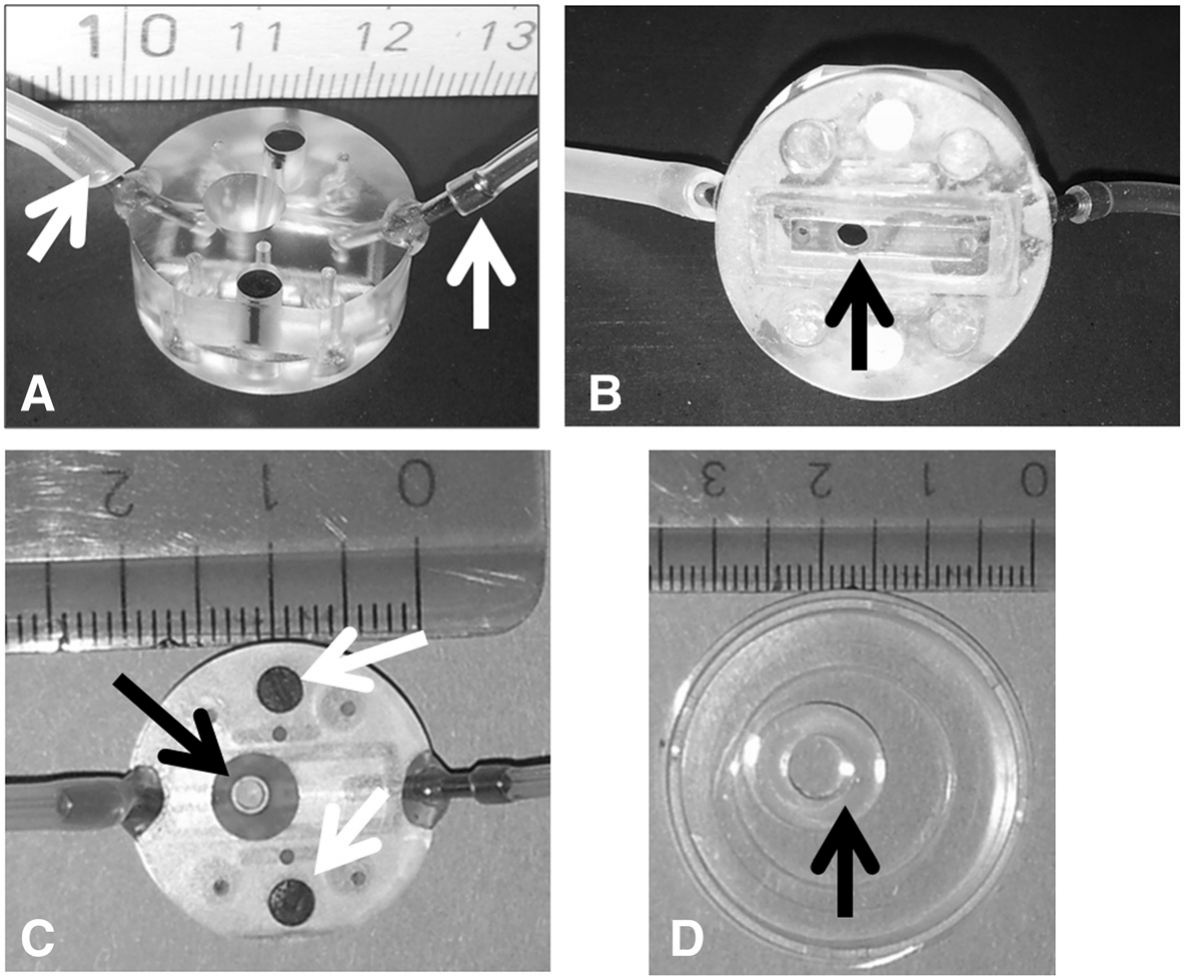
**The *in vitro *flow chamber.** The flow chamber is shown from the side (**A**), from the base (**B**) and from the top (**C**). White arrows in panel A show the inlet and outlet tubes. Black arrows in panels B and C show the field of view. A rectangle within the thin silicon mat visible in panel B surrounds the inflow and the outflow and restricts medium flow to a small chamber 2 mm wide and 0.25 mm high. White arrows in panel C show the magnets embedded into the flow chamber to fix the chamber via a metal ring opposed on the base of the culture dish. The cloning ring shown with a diameter of 0.6 cm in image D restricts the surface area of brain endothelial cells to 0.28 cm^2^. Scale is in cm.

All animal experiments for *in vitro* and *in vivo* experiments were performed in accordance with the legislation on animal welfare of the Swiss government and approved by the Kanton Bern, Switzerland. To limit the numbers of mice needed to be sacrificed for the isolation of pMBMECs, we have developed a small custom-made flow chamber (Figure 1). Growth area of pMBMECs is limited to an area of 0.28 cm^2^ by a custom-made silicon ring with a diameter of 0.6 cm (Figure 1D). Endothelial cells are stimulated with recombinant murine tumor necrosis factor alpha (TNF-α 10 ng/mL, PromoKine, Vitaris) for 16 to 20 h prior to the experiment. For optimal imaging quality, the culture dish has a hydrophilic foil-like base and excellent optical properties (μ-dish^35 mm-low^, ibidi Vitaris, Baar, Switzerland). To allow for differential interference contrast (DIC) imaging, which relies on glass or a specific DIC-compatible plastic, the field of view (FOV) is covered with glass (Figures 1B and C). The flow channel is formed from a central rectangular cut-out in a removable silicon mat. The height of the flow channel is defined by the thickness of the silicon mat and the mat is fitted onto the lower surface of the flow chamber which has inlet and outlet tubes (Figure 1B). Stable mounting of the chamber on the endothelial monolayer is achieved through two integrated magnets (Figure 1C) exerting positive magnetic pull towards a metal ring, which is placed onto the outer surface of the base of the culture dish. After the silicon ring is removed from the culture dish, the inlet tubing of the flow chamber is filled with migration assay medium (MAM) (5% calf serum, 10 mM Hepes in DMEM with glutamine) and the flow chamber is placed on the endothelial cells. Flow is applied by connecting the outlet tubing to a syringe automatically drawn up by a precision pump (Harvard Apparatus, Holliston, MA, USA). The flow rate is calculated according to the formula:

$$\tau \left(\mathrm{dyne}/cm^{2}\right)=\left(3*\mu *Q\right)/\left(2*a2*b\right)$$

[18] with μ (dynamic viscosity) = 0.083 dyne*sec/cm^2^ (DMEM, 5% calf serum at 37°C [19]);

Q (flow) = variable value to be controlled by the pump in cm^3^/sec;

a. (half height of chamber) = 0.125 mm;

b. (width of chamber) = 2 mm.

Aspiration of T cells from a reservoir via the inlet tubing is performed at 1.5 dyne/cm^2^ until the T cells appear in the field of view. T cell interaction with the endothelial surface occurs during the accumulation phase, which is started by a reduction of flow to 0.2 dyne/cm^2^. This allows settling of the T cells on the endothelial surface, which due to the size of the flow chamber only occurs under reduced shear conditions. The accumulation phase is terminated after 4 min as illustrated in Movie 1 (12 images/min, Additional file 1) and Movie 2 (3 images/min, Additional file 2); or after 8 min in Movie 3 (3 images/min, Additional file 3) by increasing the flow to 1.5 dyne/cm^2^, thus mimicking more closely physiological flow conditions within CNS post-capillary venules. Image recording in time-lapse mode is started at the beginning of the accumulation phase and continued for 15 to 30 min.

#### Microscopic equipment for computer-controlled *in vitro* live cell imaging

For microscopic imaging, the assembled flow chamber is placed on the stage of an inverted microscope (AxioObserver.Z1, Carl Zeiss, Feldbach, Switzerland) equipped with a temperature-controlled chamber (37°C). Image acquisition is performed by computer control using the AxioVision 4 software (Carl Zeiss) at a rate of 3 or 12 images per min and with a 10-fold (Objective EC “Plan-Neofluar” 10x/0,3 Ph1 M27) (Additional file 1: Movie 1), 20-fold (Objective LD “Plan-Neofluar” 20×/0,4 Korr Ph2 M27) (Additional file 3: Movie 3) or 40-fold (Objective LD “Plan-Neofluar” 40×/0,6 Korr Ph2 M27) (Additional file 2: Movie 2) magnification using a monochrome CCD camera (AxioCam MRmRev, Carl Zeiss). The size of the image (FOV) acquired with the camera depends on the microscope magnification and is 653 μm × 869 μm for 10-fold magnification, 438 μm × 329 μm for 20-fold and 215 μm × 162 μm for 40-fold.

#### Analysis of dynamic T cell interactions with the brain endothelium: T cell arrest and migratory phenotype

**Figure 2:**
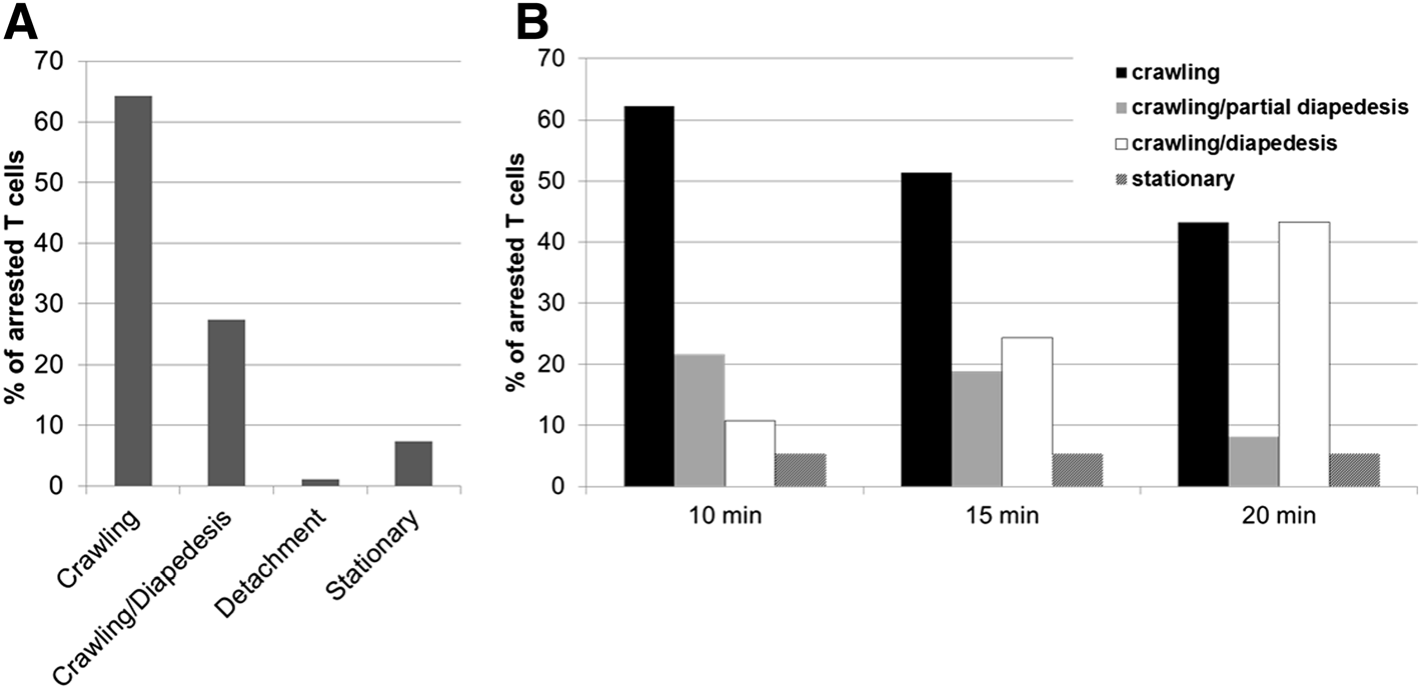
**The migratory phenotype of T cells.** Representative experiments of T cell interactions with TNF-α stimulated pMBMECs *in vitro* under flow conditions during a period of 15 minutes (2**A**) or for 3 different time periods of 10, 15 or 20 minutes (2**B**). The behavior of each arrested T cell was analyzed by eye in an offline analysis of the time-lapse videos and assigned to one category and expressed in percentage of initially arrested T cells. Arrested T cells that crawled into or out of the FOV during the recording time were excluded from the analysis. “Crawling”: T cells that polarized and crawled at least two T cell diameter distance but did not diapedese across the endothelium. “Crawling/partial diapedesis”: T cells that polarized, crawled and started but did not complete diapedesis during the indicated time period. “Crawling/Diapedesis”: T cells that polarized and crawled until they finally crossed the endothelial cell monolayer. “Detachment”: T cells that detached during the evaluation period. “Stationary”: T cells that did not polarize and remained stationary**.** 2**A**: Experiment imaged with 10x objective. A total of 64 cells were categorized. 2**B**: Experiment imaged with 20x objective. A total of 37 cells were categorized.

The dynamic interaction of T cells with the endothelium is evaluated by assigning a migratory phenotype to each T cell. To this end, each arrested T cell is assigned a digit shortly after the accumulation phase (as an example: see Additional file 3: Movie 3, at time point 8 min 20 sec). The behavior of each individual T cell is analyzed throughout the complete movie and then accordingly assigned to one category. T cells that continuously crawl are categorized as “Crawling”. T cells that diapedese after having crawled to the site of diapedesis are categorized as “Crawling/Diapedesis”, T cells that detach from the endothelium are categorized as “Detachment”. T cells that do not crawl are categorized as “Stationary” (Figure 2A). When dynamic T cell behavior with pMBMECs is imaged at higher resolution, additional categories can be defined. For example, we added a category “Crawling/partial diapedesis” describing T cells which crawled and started but did not completely diapedese during the observation time (Figure 2B). Arrested T cells that enter or leave the FOV during the recording time are excluded from the evaluation. The categories are then expressed as % of arrested T cells. To determine their crawling velocities and crawling distances, all T cells categorized as “Crawling” or “Crawling/Diapedesis” are tracked manually, using ImageJ software (National Institute of Health, Bethesda, MD, USA) using the manual tracking and chemotaxis plugins.

#### *In vitro* live cell imaging allows a detailed analysis of the dynamic behaviour of T cells adherent on the surface of BBB endothelial cells

Different *in vitro* BBB models are available for studying the cellular and molecular mechanisms of T cell migration across the BBB. We compared the migration of encephalitogenic T cells across the polyoma middle T oncogene-immortalized brain endothelial cell line, bEnd5, to primary mouse brain microvascular endothelial cells (pMBMECs) in a static two chamber-based assay as described by Röhnelt and colleagues in 1997 [20]. Although T cell adhesion to both *in vitro* BBB models was comparable, T cell diapedesis across bEnd5 was 4.5 fold more efficient when compared to migration across pMBMECs within 6 h [15]. This suggests that pMBMECs, but less so bEnd5, provide a strict barrier for T cell diapedesis as observed *in vivo*. Since the barrier characteristics of pMBMECs more closely resemble the integrity of the BBB *in vivo*, it is likely that barrier characteristics influence the cellular and molecular pathways of T cell migration across the BBB *in vitro*. Therefore we continued to study the molecular mechanisms involved in this process by employing pMBMECs derived from mice deficient for intercellular cell adhesion molecule (ICAM)-1 and ICAM-2 (ICAM-1/ICAM-2 dKO) and pMBMECs derived from wild type (wt) mice. There was a dramatic reduction of T cell diapedesis across either ICAM-1 KO or ICAM-1/ICAM-2 dKO pMBMECs when compared to wt pMBMECs [5]. A disadvantage of the static two-chamber setup is that it does not discriminate between the involvement of endothelial ICAM-1 in T cell adhesion to the BBB versus T cell diapedesis across the BBB. Therefore, we extended our experimental portfolio to an *in vitro* live cell imaging method that enables visualization of the multistep T cell extravasation across *in vitro* BBB models under conditions of physiological flow.

Although flow chambers are commercially available, we developed a small-sized flow chamber (Figure 1) suitable for a small area of cultured brain endothelial cells and for the low number of pMBMECs obtained from each isolation procedure. Using this flow chamber we visualized the dynamic behavior of encephalitogenic CD4^+^ T cells while adherent on the apical surface of pMBMECs. Whereas many T cells arrest on the surface under low shear stress, non-bound T cells are readily washed away when the shear stress is increased to physiological conditions. However, most of the T cells which resist detachment after increase of shear remain adherent throughout the remaining observation period. These T cells polarize within seconds and start crawling on the endothelial surface. Crawling occurs either continuously throughout the complete recording period, or is followed by diapedesis across the endothelial monolayer (Additional file 1: Movie 1, Additional file 2: Movie 2). The velocity of crawling on TNFα-stimulated pMBMECs is about 4 μm/min, and preferentially against the direction of flow [15]. Evaluation of the dynamic behavior of T cells while adherent to the endothelial surface is qualitatively and quantitatively analyzed such that all arrested T cells are counted and set to 100% and the 4 categories “Crawling”, “Crawling/Diapedesis”, “Detachment” and “Stationary” are expressed as fractions of initially-arrested T cells. Figure 2A shows one representative experiment using encephalitogenic CD4^+^ Th1 T cells and TNFα-stimulated pMBMECs over an observation period of 15 min. In this experiment, 64% of T cells continuously crawled, 27% crawled and diapedesed, 1% detached from the endothelium and 7% remained stationary without crawling during the observation period.

To determine how recording time affects the dynamic interaction of T cells with pMBMECs under flow *in vitro*, we analyzed the migratory phenotype at three time points: 10, 15 and 20 min (Movie 3 shows 20 min, Additional file 3). As shown in Figure 2B, 10 min recording resulted in 62% continuously crawling T cells, whereas 20 min recording reduced this to 43%. This reduction was offset by an increase in the fraction of T cells that completely diapedesed across the monolayer from 11% at 10 min to 43% after 20 min. Thus, recording times must be carefully chosen and strictly maintained during an experimental series to allow for comparable data analysis.

Employing this *in vitro* live cell imaging setup, we have compared T cell interactions on pMBMECs with those on bEnd5 cultures [15]. This showed that T cells need to crawl long distances on pMBMECs, preferentially against the direction of the flow, to find sites permissive for diapedesis. However, they readily cross a monolayer of bEnd5 cultures [5]. This supports the suggestion that the integrity of *in vitro* BBB models impacts on T cell migration across the BBB. T cell crawling against the direction of the blood flow is a unique behavior of encephalitogenic T cells when crossing inflamed spinal cord microvessels during the onset of EAE *in vivo*[6]. Hence, our *in vitro* live cell imaging setup can be used to study the cellular and molecular mechanisms involved in T cell migration into the CNS. To this end we analyzed the role of endothelial ICAM-1 and ICAM-2 in this process. Using pMBMECs from wt and ICAM-1/ICAM-2 dKO mice, we found that whereas T cell arrest on pMBMECs is mediated by endothelial ICAM-1 and VCAM-1, endothelial ICAM-1 and ICAM-2 are essential for T cell polarization and crawling on brain endothelium under flow *in vitro*[5].

Combining *in vitro* pMBMEC preparations from genetically-modified mice with live cell imaging under flow, can identify cellular and molecular mechanisms involved in the multi-step T cell migration into the CNS in the context of neuroinflammatory diseases. Observations made *in vitro*[5] can be verified *in vivo*[6]. This experimental setup can provide valuable insights into the molecular mechanisms directing transcellular or paracellular diapedesis of T cells across the BBB. It can also be used to study the multi-step migration of other immune cell subsets such as neutrophils, monocytes or CD8^+^ T cells across the BBB.

### Live cell imaging of immune cell recruitment across the BBB *in vivo*: Intravital fluorescence videomicroscopy (IVM)

#### Recipient mice and induction of active experimental autoimmune encephalomyelitis

C57BL/6 female mice, 8–12 weeks old, with an approximate body weight of 20 g were used in accordance with the local government legislation on animal welfare and experimentation. EAE was induced by sub-cutaneous immunization with 200 μg of myelin oligodendrocyte glycoprotein peptide (MOG_aa35-55_) in incomplete Freund’s adjuvant (IFA; Santa Cruz, USA) supplemented with 4 mg/mL nonviable, desiccated *Mycobacterium tuberculosis* (H37RA; Difco Laboratories, Detroit, USA) exactly as described before [10]. On day 1 and 3 post-immunization, 300 ng pertussis toxin from *Bordetella pertussis* (LuBioScience, Lucerne, Switzerland) per mouse were injected intra-peritoneally. Assessment of clinical disease score and weight of mice with active EAE was evaluated twice a day using a four-point scoring system as follows: 0, healthy; 0.5, limp tail; 1, hind leg parapesis; 2, hind leg paraplegia; and 3, hind leg paraplegia and incontinence. Mice suffering from clinical scores 0.5 (limp tail) to 2 (hind leg paraplegia), with a body weight of at least 15 g were used as recipients for IVM experiments.

#### CD8^+^ T cell isolation

CD8^+^ T cells were prepared from T cell receptor (TCR) transgenic C57BL/6 mice in which CD8^+^ T cells recognize the immunodominant MHC class I (H-2K^b^) epitope of chicken ovalbumin (SIINFEKL). Spleen and lymph nodes were collected from OT-I mice, cut in pieces and digested 30 min at 37°C in 5 mL Roswell park memorial institute (RPMI) medium supplemented with DNAse I (0.2 mg/mL; Boehringer Manheim, Germany) and liberase CI (0.4 mg/mL; Roche Applied Sciences, Switzerland). Afterwards, digested organs were crushed between 2 sterile glass slides. The resulting cell suspension was then filtered through a sterile 100 μm-nylon mesh and centrifuged for 10 min at 250 g. Cells, 7.5 × 10^6^ per 60 mm diameter petri dish, were plated in culture medium (RPMI-1640 supplemented with 10% FBS, 2 mM L-glutamine, 1 mM sodium pyruvate, 100 U penicillin-streptomycin, 0.05 mM 2-mercaptoethanol) and 50 μg SIINFEKL peptide (OVA- peptide_257-263_; Peptides international, Louisville, KY, USA) were added. Cell suspensions were incubated at 37°C in 7% CO_2_ for 5 days. On day 4, IL-2 (5 ng/mL; R&D Systems, Abingdon, UK) was added overnight in each dish. Then freshly-activated live CD8^+^ OT-I T cell blasts were isolated by Nycoprep 1.077 A (Axis-Shield, Dundee, UK) density gradient centrifugation.

#### Fluorescent labeling of T cells

After 3 to 4 days in culture, OT-I T cells were labeled with 2.5 μM Cell Tracker™ green (CMFDA; Molecular probe, Oregon, USA) in culture medium (RPMI-1640 supplemented with 10% FBS, 2 mM L-glutamine, 1 mM sodium pyruvate, 100 U penicillin-streptomycin, 0.05 mM 2-mercaptoethanol) for 45 min at 37°C in the dark. Cells were subsequently washed by adding fresh complete wash buffer (HBSS supplemented with 5% FCS and 25 mM HEPES) and centrifuged 10 min at 250 g. Excess dye was removed from the T cells by plating 5 × 10^6^ fluorescently labeled cells in a 100 mm petri dish in10 mL culture medium for 30 min at 37°C. Cell tracker™ green-labeled T cells were directly used for IVM or stored in complete medium at 37°C and 7% CO_2_ up to 6 h before use. In parallel with the spinal cord window microsurgery, 5-6 × 10^6^ of Cell tracker^TM^ green-labeled immune cells were collected and centrifuged for 10 min at 250 g. The cell pellet was then re-suspended in a small volume of NaCl 0.9% isotonic solution. Cells were counted and the volume of NaCl 0.9% isotonic solution was adjusted to obtain a cell suspension of 4 × 10^6^ cells in 300 μL. The T cell suspension was filled into a 1 mL-syringe ready for injection into the mouse circulation.

#### Microsurgical preparation of the spinal cord window

Mice were anaesthetized by subcutaneous injection of ketamine-hydrochloride/xylazine (100 mg/kg and 5.6 mg/kg respectively), followed by a subcutaneous injection of acepromazine (1.5 mg/mL). Throughout the experiment, anaesthesia of the animals was carefully monitored and, if necessary, a half dose was injected to maintain deep anaesthesia. During the surgical procedure and IVM experiment, body temperature was maintained by placing the animal on a thermo-controlled heating pad to prevent hypothermia which would influence blood supply to the brain and the hemodynamic parameters of the circulation.

Under the stereomicroscope, the right common carotid artery was catheterized in the direction of the aortic arch for systemic infusion of fluorescently-labeled T cells and 1% tetramethylrhodamine isothiocyanate (TRITC)-conjugated Dextran used as a plasma marker. Afterwards, the animal was turned to the prone position and the head was placed in a stereotactic holder. The midline skin of the neck was incised for 2–3 cm and the paravertebral musculature was dissected from the cervical spine processes and retracted laterally by the use of 4–0 threads, exposing the vertebral lamina. A laminectomy was then carried out from C7 to C2 and the dura mater over the spinal cord was removed avoiding any trauma to the microvasculature and underlying spinal cord parenchyma. The preparation was then covered with a transparent plastic membrane to prevent dehydration and access of ambient O_2_ to the exposed tissue.

#### Intravital fluorescence videomicroscopy (IVM)

**Figure 3:**
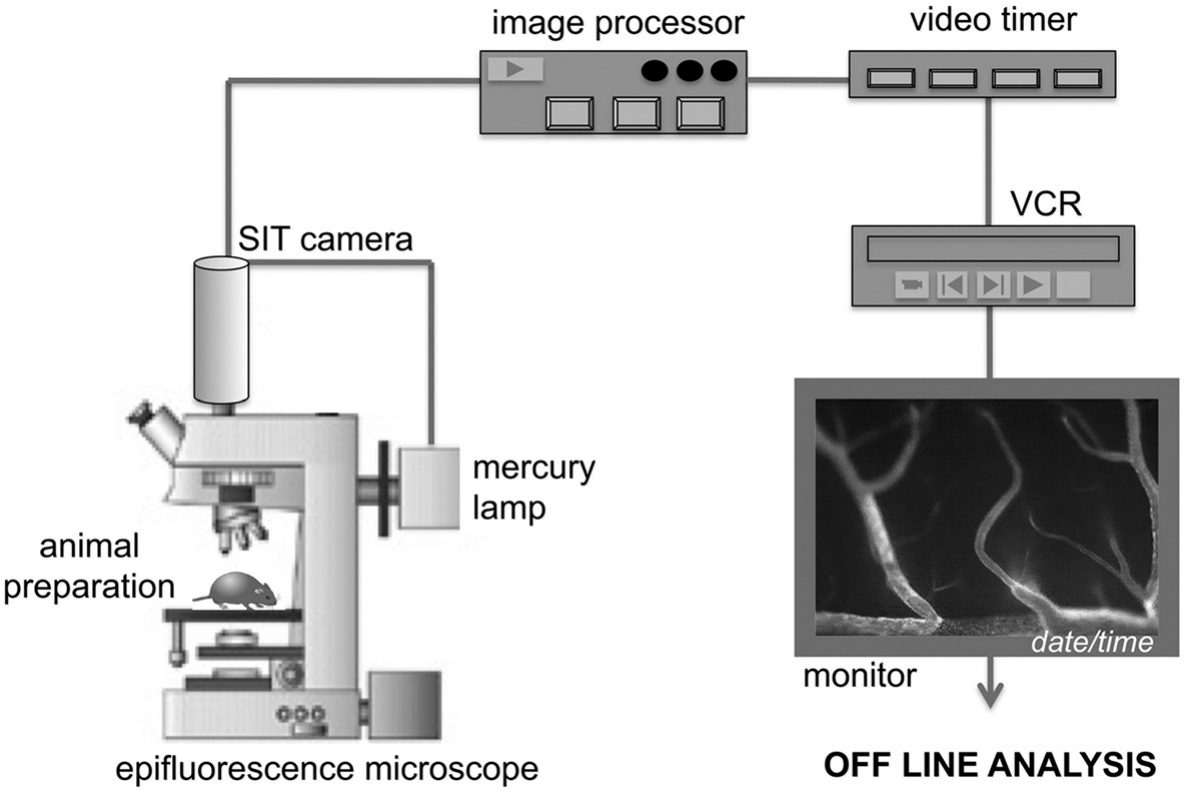
**Experimental setup of the intravital fluorescence videomicroscopy workstation.** The animal preparation under anesthesia is placed under an epifluorescence microscope, coupled with a mercury lamp connected to a low-light-imaging silicon-intensified target (SIT) camera that includes an image processor, associated videotimer, a digital videocassette recorder (VCR) and a video monitor. For later off-line analysis, real time videos were recorded using a digital videocassette.**A:** Evaluation of the initial contact fraction (%) of OT-I CD8^+^ T cells with the post capillary venules (20–60 μm diameter) of the spinal cord microvasculature of mice with EAE **B:** Shows the evaluation of capture and rolling fractions (%) of OT-I CD8^+^ T cells with the post capillary venules (20–60 μm diameter) of the spinal cord white matter microvasculature of mice afflicted with MOG_35-55_-induced EAE.

The animal remaining within the stereotactic head holder was transferred to the stage of the inverted fluorescence microscope (Figure 3). IVM was performed by epi-illumination techniques using a custom-made Mikron IVM500 microscope (Mikron Instruments, San Marcos, CA, USA) coupled with a 50 W mercury lamp (HBO 50 microscope illuminator, Zeiss, Switzerland) attached to combined blue (exciter 455DF70, dichroic 515DRLP, and emitter 515ALP) and green (exciter 525DF45, dichroic 560DRLP, and emitter 565ALP) filter blocks. The microscope is connected to a low-light-imaging silicon-intensified target (SIT) camera (Dage-MTI Inc., Michigan city, IN, USA) coupled with a Trinitron® color video monitor (Sony, Switzerland) and a videotimer (MicroImage Video Systems, Boyertown, USA). For later real time off-line analysis, images were recorded using a digital videocassette recorder (VCR) (Figure 3). Observations were made using a × 4, ×10 and × 20 long-distance objectives (Zeiss, Switzerland), resulting in × 80, ×215 and × 440 magnifications, respectively.

First, the microvasculature of the spinal cord was observed within the green light epi-illumination (×4 objective) by intra-carotid injection of the pre-warmed fluorescent plasma marker TRITC-conjugated Dextran (1%, MW = 155,000; Sigma-Aldrich, Switzerland) in 0.9% isotonic NaCl. The spinal cord is divided into two parts by the middle dorsal vein, delineating a top and bottom half of the entire window. On both sides, capillaries and post-capillary venules draining into the middle dorsal vein can readily be visualized. Between 4 and 6 stepwise FOVs per animal could be delineated on each side of the spinal cord window (10× objective). Using the blue light epi-illumination (10× objective), 4 × 10^6^ Cell Tracker™ green-labeled activated OT-I CD8^+^ T cells were slowly infused in 3 aliquots of 100 μL and were directly observed within the spinal cord microcirculation where they initiated contact with the inflamed spinal cord white matter endothelium. For each injection of 100μL, a different FOV was recorded for a minimum of one minute in order to observe sufficient CD8^+^ T cells interacting with the endothelium for later off-line analysis. After the infusion of each aliquot, the arterial catheter was flushed with 60 to 80 μL pre-warmed isotonic 0.9% NaCl to guarantee all cells were injected. At different time points after cell injection (10 min, 30 min and 1 h), all fields of view of the spinal cord window were sequentially scanned and recorded for further evaluation of the number of permanently adhering fluorescent CD8^+^ T cells. At the end of the recording period, animals were sacrificed.

#### Targeting of cell surface adhesion molecules on BBB endothelium

In order to evaluate the involvement of a specific adhesion molecule or its ligand in T cell trafficking across the spinal cord microvasculature endothelium *in vivo*, activated T cells or the BBB endothelium were pre-treated with function-blocking antibodies. To this end, 4 × 10^6^ Cell Tracker^TM^ green-labeled CD8^+^ T cell blasts in 300 μL isotonic 0.9% NaCl solution were incubated with 120 μg blocking monoclonal antibody (mAb) directed against a specific adhesion molecule for 20 min prior to their injection into the bloodstream. The use of antibodies *in vivo* requires endotoxin-free antibody preparations and appropriate isotype controls. Using non-blocking antibodies from the same isotype as the blocking mAb, ensures against nonspecific side effects mediated by the Fc portions of the immunoglobulins. Control antibodies specific for molecules expressed on the surface of circulating immune cells or on the BBB endothelium, which do not interfere with T cell trafficking are preferable over non-binding irrelevant isotype control antibodies remaining in the circulation. In this study, rat-anti-mouse α4-integrin (PS/2), rat anti-mouse α4β7 integrin (DATK-32), and rat anti-mouse β7 integrin (Fib 504) were used and obtained from serum-free hybridoma culture supernatants. Endotoxin levels, determined using Endosafe test (Charles River Laboratories, Sulzfeld, Germany), were below detection level. Endotoxin-free rat IgG2b was used as isotype control.

#### Quantitative analysis of the IVM data

##### Initial contact of circulating T cells within post-capillary venules of the spinal cord white matter in mice with active EAE

From each observed post-capillary venule (diameter = 20–60 μm), the percentage of T cells initiating contact with the BBB endothelium as observed by IVM was determined at the time point of cell injection. The total number of T cells was injected in 3 aliquots and 1 FOV was visualized for each injection. Thus the initial interaction of circulating T cells could be analyzed in a substantial number of spinal cord post-capillary venules per animal. The number of T cells (> 10 cells/min) rolling along the vessel wall or captured (abruptly arrested without any preliminary rolling step) was counted per post-capillary venule, and related to the total number of fluorescent circulating T cells (total cellular flux, TFx) passing through the vessel during one minute. The rolling fraction (RF) or the capture fraction (CF) was calculated and the total initial contact fraction (ICF) calculated from the sum of RF and CF (summarized in Table 1). Both rolling and capture events were confirmed by calculating the critical velocity in μm.s ^-1^ (V_crit_). V_crit_ is the velocity of an idealized cell traveling along, but not interacting with the vessel wall. It can be derived from the parabolic velocity profile of the circulation in the microvessel, as follows:

$$V_{\mathrm{crit}}=V_{\mathrm{blood}}\times \left(D_{L}/D_{V}\right)\times \left[2-\left(D_{L}/D_{V}\right)\right]$$

in which D_L_ and D_V_ correspond to the diameter (mm) of the leukocyte and the diameter of the post-capillary venule, respectively, and V_blood_ corresponds to the mean blood flow velocity (summarized in Table 1). Any leukocyte circulating below V_crit_ was considered as an interacting cell rolling along the vessel wall, whereas any cell traveling above V_crit_ was defined as a non-interacting cell [21,22]. Statistics using Mann–Whitney *U*-Test to compare 2 variables and Kruskall-Wallis to compare more than 2 variables were then performed.

**Table 1:** **Parameters analyzed by intravital microscopy (modified from**[22]**)**

**Parameter**	**Unit**	**Formula**
Total Cellular Flux (TFx)	min^-1^	Number of T cells that pass through a vessel during a given observation period
Initial Contact Flux (ICFx)	min^-1^	Number of immune cells that interact, either by rolling or getting captured to, with the vessel wall during an observation period
Initial Contact Fraction (ICF)	%	ICF = ICFx/TFx × 100
Rolling Flux (RFx)	min^-1^	Number of immune cells that roll along the vessel wall during an observation period
Rolling Fraction (RF)	%	RF = RFx/TFx × 100
Capture Flux (CFx)	min^-1^	Number of immune cells that are captured to the vessel wall during an observation period
Capture Fraction (CF)	%	CF = CFx/TFx × 100
Firm Adhesion/Field Of View (FOV)	none	Number of firmly adherent T cells per one field of view (FOV) within the spinal cord window

##### Firm adhesion of T cells within inflamed spinal cord post-capillary during EAE

Firmly adherent T cells were identified as fluorescent cells that stick to the vessel wall without moving or detaching. Trapped T cells within the capillary network, were defined as cells that do not move and clearly obstruct the capillary lumen, resulting in blood flow stasis. The permanent adhesion of T cells at 10 min, 30 min and 1 h after infusion was expressed as the number of adherent and trapped T cells per field of view (FOV) observed with the × 10 objective [23]. As 4–6 FOVs could be identified on each side of the spinal cord window, all calculations of firmly adherent T cells per FOV from different mice were grouped to calculate the mean +/− standard deviations for each animal. Statistics using Mann–Whitney *U*-Test to compare 2 variables and Kruskall-Wallis to compare more than 2 variables are then performed.

#### Contribution of a4β1-versus a4β7-integrin in the interaction of CD8^+^ T cells with the inflamed BBB *in vivo*

Blocking T cell entry into the CNS with the humanized anti-α4 integrin antibody, natalizumab, has proved efficient in the treatment of relapsing-remitting multiple sclerosis [11]. However, natalizumab is associated with an increased risk for developing progressive multifocal leukoencephalopathy, a fatal disease of the CNS caused by JC virus infection of oligodendrocytes [24]. This observation suggests that therapeutic targeting of α4-integrins may ultimately impair immunosurveillance of the CNS by cytotoxic CD8^+^ T cells.

**Figure 4:**
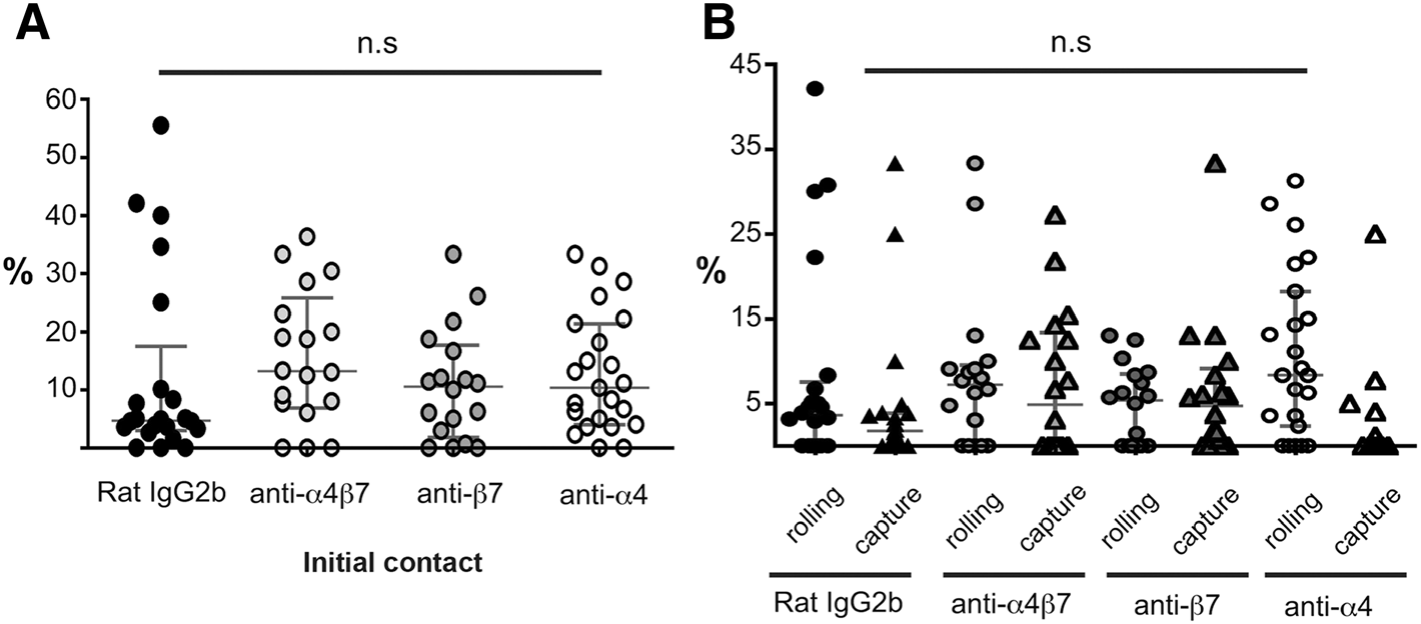
**Quantification of OT-I CD8**^**+**^**T cell interactions with the spinal cord microvasculature *in vivo. *A:** Evaluation of the initial contact fraction (%) of OT-I CD8^+^ T cells with the post capillary venules (20–60 μm diameter) of the spinal cord microvasculature of mice with EAE. Each dot represents 1 venule. All values show the median with the interquartile range of n = 22 analyzed post-capillary venules from 3 mice for rat IgG2b condition, n = 18 analyzed post-capillary venules from 5 mice for anti- α4β7 condition, n = 18 analyzed post-capillary venules from 6 mice for anti- β7 condition and n = 23 analyzed post-capillary venules from 4 mice for anti-α4 condition. **B:** Shows the evaluation of capture and rolling fractions (%) of OT-I CD8^+^ T cells with the post capillary venules (20–60 μm diameter) of the spinal cord white matter microvasculature of mice afflicted with MOG_35-55_-induced EAE. N = 22 analyzed post-capillary venules from 3 mice for rat IgG2b condition, n = 18 analyzed post-capillary venules from 5 mice for anti- α4β7 condition, n = 18 analyzed post-capillary venules from 6 mice for anti-β7 condition and n = 23 analyzed post-capillary venules from 4 mice for anti-α4 condition. Statistical significance was determined by the Mann–Whitney *U*-Test.

**Figure 5:**
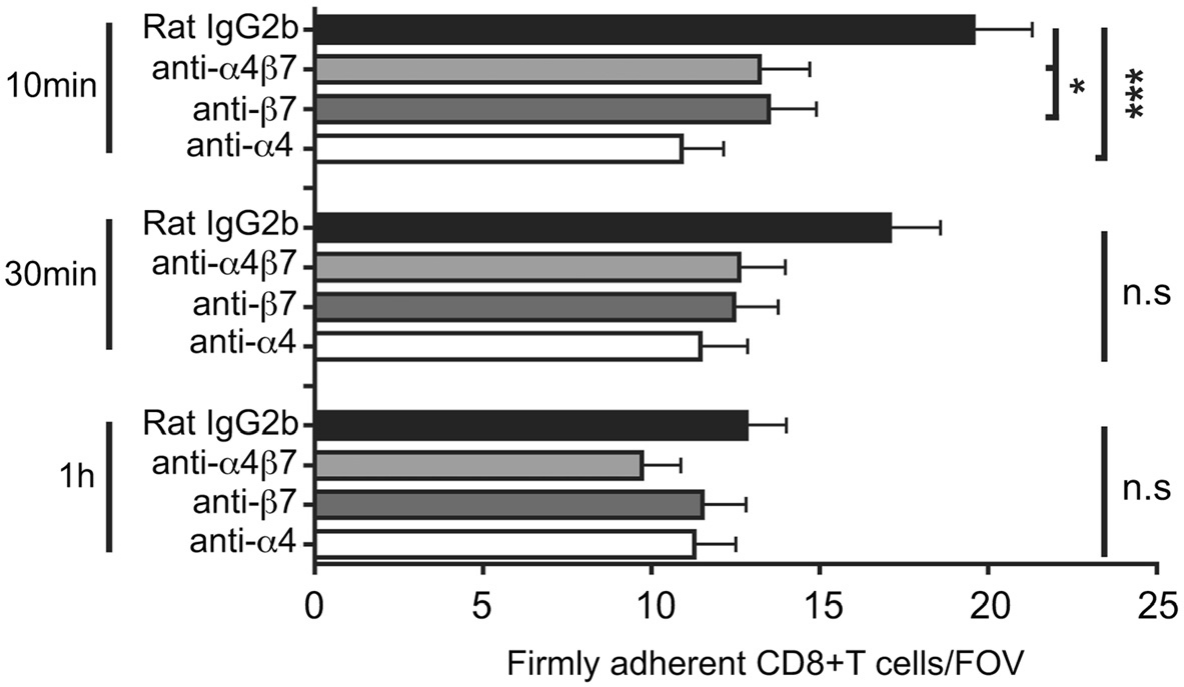
**Quantification of OT-I CD8**^**+**^**T cell firm adhesion to the post capillary venules of the spinal cord microvasculature of C57BL/6 mice during EAE.** Permanently adhering OT-I T cells were counted 10 min, 30 min and 1 hour after cell infusion. Each dot represents the number of adherent OT-I T cells/field of view (FOV). The numbers of mice analyzed at t = 10 min for each condition was n = 8 for rat IgG2b, n = 6 for anti-α4β7, n = 6 for anti-β7 and n = 8 for anti-α4. At t = 30, n = 8 for rat IgG2b, n = 6 for anti-α4β7, n = 6 for anti-β7 and n = 4 for anti-α4. At time t = 1 h, the number of mice was n = 7 for rat IgG2b, n = 5 for anti-α4β7, n = 5 mice for anti-β7 and n = 5 for anti-α4. Data are presented as mean values +/− standard deviation (SD). Mann–Whitney *U*-Test was used for comparisons between different data sets. Asterisks indicate significant differences (*P < 0.05, and ***P < 0.005), n.s.: not significant.

To investigate if CD8^+^ T cells use molecular mechanisms similar to CD4^+^ T cells to migrate across the BBB *in vivo,* we studied the interaction of CD8^+^ OT-I T cells with the inflamed spinal cord white matter microvasculature in C57BL/6 mice during EAE to determine if CD8^+^ T cells also use α4β_1_- but not α4β7-integrins to adhere to the inflamed BBB as previously shown for CD4^+^ T cells [25,26]. The purity of CD8^+^ OT-I T cell preparations was confirmed by FACS staining, which demonstrated that 95% of the OT-I T cell blasts stained positive for CD8, an acceptable purity for performing IVM (data not shown). Prior to their infusion into the circulation of the recipient mouse, fluorescently-labeled OT-I T cell blasts were pre-treated with integrin blocking or control antibodies (480 μg Ab/ 4 × 10^6^ OT-I T cells/ 400 μl with the exception of DATK-32, which was used at 960 μg/ 4 x 10^6^ OT-I T cells/ 400 μl due to its low affinity). Following the visualization of the spinal cord vascular system by injection of TRITC-dextran, OT-I T cells were systemically infused via the right carotid artery and their interaction with the spinal cord microvasculature observed and recorded in real time (Figure 3, Additional file 4: Movie 4 and Additional file 5: Movie 5). The initial contact (rolling and capture) and firm adhesion of OT-I T cells to the spinal cord vasculature were evaluated by off-line frame-by-frame video analysis. The following conditions were studied: rat IgG2b used as a control antibody, PS/2 (anti-α4 subunit), DATK-32 (anti-α4β7 integrin) and Fib 504 (anti-β7 subunit). Upon systemic infusion, activated OT-I T cells were observed to pass through the spinal cord microvessels and initiate contact with the inflamed CNS endothelium (Additional file 4: Movie 4). Contact initiation was mediated either by OT-I T cells rolling with reduced velocity along the vascular wall or to a lesser degree by capture, i.e. an abrupt arrest of the CD8^+^ T cells on the vascular wall. Pre-treatment of OT-I T cells with either isotype control mAb or blocking antibodies against α4-, β7- or α4β7- integrins showed no effect on their intrinsic abilities to initiate contact with the inflamed BBB endothelium (Figure 4A), either by rolling or capture to the spinal cord microvasculature wall (Figure 4B). To determine whether the initial contact of OT-I T cells resulted in arrest and firm adhesion to the inflamed microvasculature (Additional file 5: Movie 5), the number of OT-I T cells permanently adhering within microvessels at different time points (10 min, 30 min and 1 h) after T cell infusion for each tested condition was measured (Figure 5). Ten minutes after infusion, the inhibition of α4-integrins resulted in a 50% reduction of the firm adhesion of OT-I T cells to the microvasculature when compared to IgG2b isotype control treatment, whereas blocking of α4β7- or β7-integrins only reduced the adhesion of OT-I T cells by 30%. These data suggested that both α4-integrins mediate OT-I adhesion to the inflamed spinal cord microvasculature. Interestingly, the involvement of α4-integrins in mediating OT-I T cell adhesion to the inflamed BBB was only transient, as at the later times adhesion of OT-I T cells was no longer inhibited by the presence of α4-integrin blocking antibodies. At these times there were lower numbers of OT-I cells firmly adhering under control conditions. These results suggest that during EAE, activated CD8^+^ T cells interact with the inflamed BBB. In contrast to CD4^+^ T cell blasts, CD8^+^ T cells are able to initiate contact and to maintain stable adhesion to the inflamed BBB independent of α4-integrins [10,25].

#### Statistical analysis

All statistical analysis were performed using the GraphPad Prism software (version 5.00, GraphPad Software, CA, USA). Data are presented as mean values +/− standard deviation (SD). Mann–Whitney *U-*Tests were used for comparisons between different data sets. Asterisks indicate significant differences (*P < 0.05, **P < 0.01 and ***P < 0.005).

## Discussion

Investigation of the cellular and molecular mechanisms of T cell migration across the BBB in the context of MS has become possible with the development of live cell imaging approaches that record the dynamic interaction with the BBB during EAE. Using a flow chamber setup for brain endothelial cell cultures or a microsurgical window for observation of the spinal cord microvasculature, has enabled the study of dynamic T cell interactions with the BBB under physiological flow both *in vitro* and *in vivo*.

The *in vitro* flow chamber with time-lapse live cell imaging has been used to study the post-arrest dynamic behavior of encephalitogenic CD4^+^ T cells on the inflamed BBB under flow conditions. The cellular and molecular events underlying the multi-step T cell extravasation across the inflamed BBB *in vitro* have been studied and the functions of different endothelial adhesion molecules in mediating CD4^+^ T cell arrest, versus polarization and crawling were delineated. These experiments underline the active role of the BBB endothelium in controlling T cell extravasation during immunosurveillance and inflammation. Results *in vitro* have been confirmed *in vivo* by two recent studies investigating T cell extravasation across the spinal cord microvasculature during EAE by two-photon IVM [6,27], which showed that T cells crawl long distances against the direction of the blood-flow on the spinal cord endothelial surface to find a site permissive for diapedesis using the molecular mechanism found in our studies [5].

Using high resolution *in vitro* imaging, we are studying the cellular and molecular mechanisms involved in T cell diapedesis across the BBB under physiological flow to determine if T cells breach the BBB via a transcellular or paracellular route. With pMBMEC preparations of gene-targeted mice and fluorescent-tagged adhesion and junctional molecules, it will be possible to distinguish the molecular events in these processes.

It is important to note that although the flow chamber set-up described here is suitable to study the entire multi-step T cell extravasation across the BBB, the combination with time lapse imaging does not allow fast movements as observed during T cell tethering or rolling on the BBB to be recorded. Whereas T cell rolling along the BBB occurs with velocities of several hundreds of μm per second, T cell polarization and crawling events as described here are much slower and occur at velocities of several μm per minute. Thus investigation of T cell tethering and rolling using such an *in vitro* flow chamber requires real time imaging at 20 images per second, minimum, or even more than 30 images per second.

In contrast, the IVM real time imaging method described here is optimal to study the initial interaction (rolling/capture), the arrest and the firm adhesion of T cells within the spinal cord microvasculature under physiological flow conditions *in vivo*. Observation times of one minute suffice to study the initial T cell interaction with the spinal cord microvasculature *in vivo* and therefore avoid phototoxic effects on the vasculature. Similarly, one minute video sequences of the different FOVs at defined time points after systemic T cell infusion will enable study of T cell adhesion to the BBB *in vivo* over extended times. Due to the short observation times necessary, we have previously used this imaging approach to successfully study the interaction of human T cells with the spinal cord microvasculature during EAE *in vivo* in immunocompetent mice, since human integrins engage with mouse endothelial ligands comparable to the human endothelial ligands [10]. In this xenogeneic approach we showed that the anti-α4-integrin antibody natalizumab, used for the treatment of relapsing-remitting MS, specifically blocks T cell adhesion, but not rolling, during EAE *in vivo*[10].

The spinal cord window described here is located at the level of cervical spinal cord (C7-C5) and allows direct visualization of both the spinal cord leptomeningeal and white matter microvessels under physiological conditions [9]. During EAE, when inflammatory reactions increase the depth of the leptomeningeal space on the surface of the spinal cord, visualization of white matter microvessels is limited due to the limitation of epifluorescence technique which has a tissue penetration of 50-70 μm. In contrast, the lumbar spinal cord window usually employed for live cell imaging in the spinal cord only allows for observation of the leptomeningeal blood vessels, even when employing 2P-IVM with deeper penetration into the tissue [6]. This might be due to the differences in the angioarchitecture at the different levels of the spinal cord.

The IVM approach introduced here can certainly be extended to study the interaction of immune cell subsets other than T cells with the spinal cord microvasculature *in vivo*. Using the same experimental approach as described for T cells we were able to show that immature dendritic cells migrate into the CNS during EAE and use α4-integrins to adhere to the inflamed spinal cord microvasculature *in vivo*[28]. A critical prerequisite to study the interaction of a given immune cell subset with the spinal cord microvasculature using the IVM method described here, is to obtain a highly purified population of the cells of interest. This is due to the fact that only a limited number of cells infused into the systemic blood gain access to the observation window of the spinal cord and even less cells (about 10–20 fluorescent immune cells per field of view (FOV) with 5–6 FOV per spinal cord window) are expected to interact with the endothelium of the exposed spinal cord window microvasculature.

To study CD8^+^ T cell interaction with the spinal cord microvasculature during EAE we have therefore decided to first investigate CD8^+^ T cells from a TCR transgenic OT-I mouse. This allowed for a homogeneous ovalbumin-specific T cell activation *in vitro* which resulted in a population of activated CD8^+^ T cells with more than 95% purity. Here we demonstrated that activated CD8^+^ T cells successfully interact with the inflamed spinal cord microvessels during EAE. We therefore asked whether α4-integrins, which are essential for the migration of CD4^+^ T cells across the BBB, play any role in the multi-step CD8^+^ T cell extravasation across the BBB *in vivo*. Here we found that α4β7-, β7- or α4-integrins are not required for the rolling and capture of CD8^+^ T to the inflamed spinal cord white matter microvasculature. This is in accordance with our previous findings demonstrating that β1-integrin deficient CD4^+^ and CD8^+^ T cells have no defect in capturing and rolling on the inflamed BBB during EAE [25] and that natalizumab fails to interfere with the rolling and capturing of human T cells to the inflamed spinal cord microvessels during EAE [10]. Interestingly, although we initially saw a contribution of α4-integrins in mediating the firm adhesion of CD8^+^ T cells to the inflamed spinal cord microvasculature, this effect was lost mainly due to the low number of firmly adhering CD8^+^ T cells observed in the control group over time. These observations therefore suggest that stable adhesion of CD8^+^ T cells to the inflamed BBB *in vivo* does not critically rely on α4-integrins. Considering our previous observation that β1-integrin deficient CD8^+^ T cells fail to enter the CNS parenchyma during EAE [25], we propose that β1-integrin mediated adhesions might be critical at a later step, namely in CD8^+^ T cell crossing the endothelial basement membrane.

Although the IVM approach described here allows for live cell imaging of immune cell interactions with the spinal cord microvasculature under physiological and pathological conditions, some limitations apply due to the fact that single-photon excitation used in conventional epifluorescence video microscopy requires short wavelength and therefore high energy excitation light. This results in a disadvantageous high risk of phototoxicity and the restriction of imaging depth to 70 μm. These limitations have been overcome by the introduction of two-photon IVM (2P-IVM) which enables deep tissue penetration with less absorption or scattering of fluorescent light than conventional IVM (for details see [29]). 2P-IVM has a CNS tissue penetration of 800–1000 μm [30]. It yields time-lapse videos with a high 3D resolution allowing observation of immune cell interactions with the spinal cord microvasculature over a long time period. It is therefore suitable for observing the slow post-arrest immune cell interactions with the spinal cord microvasculature such as T cell polarization, crawling and diapedesis taking place at velocities about 10 μm/min *in vivo*[6]. In contrast, 2P-IVM is not suited to investigate the molecular mechanisms involved in the fast initial T cell interaction steps with the BBB *in vivo* taking place at velocities of about 40–100 μm/s.

In summary, combining state-of-the-art live cell imaging approaches with *in vitro* BBB models and sophisticated surgical window preparations for *in vivo* observation of the CNS microvasculature, provides a powerful experimental approach to identify the molecular mechanisms employed by the BBB to control immune cell trafficking into the CNS. The identification of some of these traffic signals has proved to be of clinical importance as blocking these molecules reduces the migration of pathogenic immune cells into the CNS and proven beneficial for the treatment of MS. In contrast, induction or enhancement of immune cell trafficking signals on the BBB could be beneficial for the treatment of CNS infections or neoplasia.

## Abbreviations

BBB: Blood brain barrier; CF: Capture fraction; CNS: Central nervous system; DIC: Differential interference contrast; DMEM: Dulbecco’s modified Eagle’s medium; EAE: Experimental autoimmune encephalomyelitis; FACS: Fluorescence activated cell sorting; FBS: Fetal bovine serum; FOV: Field of view; GPCR: G-protein coupled receptor; HBSS: Hank’s balanced salt solution; Hepes: N-2-hydroxyethylpiperazine-N’-2-ethanesulfonic acid; ICF: Initial contact fraction; ICAM: Intercellular adhesion molecule; IVM: Intravital fluorescence videomicroscopy; mAb: Monoclonal antibody; MAM: Migration assay medium; MHC: Major histocompatibility complex; MOG: Myelin oligodendrocyte glycoprotein; MS: Multiple sclerosis; PLP: Proteolipid protein; pMBMECs: Primary mouse brain microvascular endothelial cells; RF: Rolling fraction; RPMI: Roswell park memorial institute medium; SIT: Silicon-intensified target; TFx: Total cellular flux; TCR: T cell receptor; TNF-α: Tumor necrosis factor-α; TRITC: Tetramethylrhodamine isothiocyanate; VCAM-1: Vascular cell adhesion molecule; VCR: Video cassette recorder; Wt: Wild type.

## Competing interests

The authors declare that they have no competing interests.

## Authors’ contributions

BE designed the live cell imaging studies, developed the basic concept of the article and has written parts of the article. RL developed the *in vitro* flow chamber assay and significantly contributed to the rational of the experiments. CC and RL performed the experiments, made the Figures and Movies and have written parts of this article. All authors have read and approved the manuscript.

## Supplementary Material

Additional file 1 **Movie 1.** Shear resistant arrest, polarization, crawling and diapedesis of CD4^+^ T cells on and across TNF-α stimulated wt pMBMECs under flow (low magnification). CD4^+^ T cells were perfused over TNF-α stimulated pMBMECs under low shear (0.1 dyn/cm^2^) (upper timer). After 4 min, flow was increased to physiological shear stress (1.5 dyne/cm^2^) (lower timer). The number of arrested CD4^+^ T cells constantly increased during the accumulation phase. Physiological shear washed away unbound T cells. Only few arrested CD4^+^ T cells detached from the endothelial surface whereas the majority of CD4^+^ T cells either continuously crawled or crawled and diapedesed through the endothelium. Phase-contrast bright T cells crawl on the apical surface of the endothelium, whereas phase-contrast dark T cells crawl beneath the endothelium. Direction of flow is from left to right. Objective 10x (Objective EC “Plan-Neofluar” 10x/0,3 Ph1 M27), phase-contrast illumination at 12 images per min, recording time 19 min. Movie at 12 images per sec, field of view 653 μm x 869 μm. Click here for file

Additional file 2 **Movie 2.** Shear-resistant arrest, polarization, crawling and diapedesis of CD4^+^ T cells on and across TNF-α stimulated wt pMBMECs under flow (high magnification). The experimental setup was identical to that described in Movie 1. Images were taken with a 40x objective (Objective LD “Plan-Neofluar” 40x/0,6 Korr Ph2 M27) under differential interference contrast illumination at 3 images per min; recording time 14.5 min. Movie at 8 images per sec; field of view 215 μm x 162 μm. Click here for file

Additional file 3 **Movie 3.** Shear-resistant arrest, polarization, crawling and diapedesis of CD4^+^ T cells on and across TNFα stimulated wt pMBMECs under flow (high magnification). Movie corresponds to the evaluation shown in Figure 2b. The experimental setup was identical to that described in Movie 1. Flow increase to physiological shear stress (1.5 dyne/cm^2^) was at 8 min (lower timer). Numbers placed on T cells visible on one frame of the movie (lower timer = 40 sec) were assigned for identification of each individual T cell. Images were taken with a 20x objective (Objective LD “Plan-Neofluar” 20x/0,4 Korr Ph2 M27) under phase-contrast illumination at 3 images per min; recording time 21 min; movie taken at 6 images per sec; field of view 438 μm x 329 μm. Click here for file

Additional file 4 **Movie 4.** Initial contact of CD8^+^ T cells with the spinal cord microvasculature during EAE. At the beginning, the inflamed spinal cord microvasculature is visualized by TRITC-Dextran in the circulation of a C57/BL6 mouse affected with EAE within one field of view (FOV) at x10 objective. The spinal cord white matter microvasculature is visualized above the large spinal cord collecting vein, always seen at the bottom of each video frame. After switching the fluorescence filter on the microscope, the infusion of 1 aliquot (1.3 x 10^6^ cells/100μL) of CellTracker green CD8^+^ T cells, pretreated for 20 min with rat IgG isotype control, is started and T cells can be observed either passing through the corresponding vascular beds or interacting as rolling or capturing to the microvasculature in real time (objective x10) within one FOV. Click here for file

Additional file 5 **Movie 5.** Firm adhesion of CD8^+^ T cells to the spinal cord microvasculature during EAE. This movie first shows the entire spinal cord window (x 4 objective) 10 minutes after infusion of the total amount of CD8^+^ T cells (4 x 10^6^ cells), then the scanning of several fields of view within the entire spinal cord window to quantify those T cells that firmly adhere to the inflamed spinal cord microvasculature (x 10 objective). Click here for file
